# Cardiovascular Events Among Women with Premature Ovarian Insufficiency: A Systematic Review and Meta-Analysis

**DOI:** 10.31083/j.rcm2407193

**Published:** 2023-07-04

**Authors:** Samira Behboudi-Gandevani, Ellen Christin Arntzen, Britt Normann, Tommy Haugan, Razieh Bidhendi-Yarandi

**Affiliations:** ^1^Faculty of Nursing and Health Sciences, Nord University, 8049 Bodø, Norway; ^2^Faculty of Nursing and Health Sciences, Nord University, 7629 Levanger, Norway; ^3^Department of Biostatistics, University of Social Welfare and Rehabilitation Sciences, 1985713871 Tehran, Iran

**Keywords:** cardiovascular events, premature ovarian insufficiency, systematic review and meta-analysis

## Abstract

**Background:**

It is well documented that menopause is linked to an increased risk of cardiovascular (CV) events; however, the results of studies focusing on the association between premature ovarian insufficiency (POI) and the risk of CV events are controversial. The aim of this systematic review and meta-analysis was to assess the risk of CV events among women with POI compared to women with menopausal aged 50–54 years.

**Methods:**

A systematic literature search of PubMed (including Medline), Scopus, and Web of Science was conducted from 1990 to 2022 to retrieve observational studies published in English-language. The studies’ quality was assessed using structured standard tools. Primary-outcome was the pooled risk of the composite outcome of CV events.

**Results:**

We included 16 studies involving 40,549 women who suffered from POI and 1,016,633 women as controls. After adjustment for hormone therapy, the pooled risk of composite outcome of CV events and coronary heart disease, among women with the POI was significantly 1.3 (Pooled-adjusted hazard ratio (HR) = 1.35, 95% CI: 1.06–1.63, $I^{2}$: 0%) and 1.4 (Pooled adjusted HR = 1.42, 95% CI: 1.17–1.66, $I^{2}$: 0%) fold higher than women with menopausal age 50–54 years. There was no difference between the groups regarding the risk of stroke and death due to CV events between two groups. There was not sufficient data for pooled analysis of other specific CV events.

**Conclusions:**

In conclusion, POI is associated with an increased risk of CV events, particularly coronary heart disease. Our findings extend prior work with data supporting POI as a risk-enhancing factor for CV events. However, more studies are needed to confirmed these findings.

## 1. Introduction 

Worldwide cardiovascular disease (CVD) accounts for one of the highest proportions of non-communicable diseases, is a leading cause of morbidity and a major contributor to disability [1, 2, 3]. Cardio-metabolic disturbances, genetic, behavioral, environmental, and psycho-social risk factors are major drivers of CVD [1, 4, 5].

It is well documented that there are substantial differences between men and women in the prevalence and burden of different CVDs [6]. In this respect, there are a number of clinical conditions unique to women that have been described as female specific factors which increase CVD risks [7, 8, 9, 10]. Likewise, menopause has been related to an increased risk of CVDs in many studies [11, 12]. The exact underlying etiology is not fully understood, but it may be strongly attributed to estrogen deprivation [13]. This hormone, as the main sex steroid hormone, has some cardioprotective mechanisms such as increased angiogenesis, and vasodilatation and also may reduce fibroblast proliferation and antiapoptotic properties [14]. The average age at the onset of menopause is around 51 years [15], however, in 3–4% of all women, it occurs before the age of 40 years, which is called premature ovarian insufficiency (POI) [16]. The preliminary studies reported that POI could be an independent risk factor for cardiovascular mortality and morbidity [17, 18].

The aim of this systematic review and meta-analysis was to assess the risk of developing cardiovascular events among women with POI compared to women with age at menopausal 50–54 years.

## 2. Methods 

This systematic review and meta-analysis was performed by the Preferred Reporting Items for Systematic Reviews and Meta-Analyses (PRISMA) [19], to achieve the following objectives: (i) to study the pooled risk of developing the composite outcome of all types of cardiovascular events, regardless of the type of events among women with POI defined as menopause <40 years compared to menopause aged 50–54 years; (ii) to study the pooled risk of developing specific cardiovascular events including coronary heart disease, stroke, heart failure, heart valve disease, pulmonary hypertension, chronic hypertension and death due to any cardiovascular events among women with POI compared to women with age at menopause of 50–54 years.

The review question was formulated using the PICO (population, intervention/index, control, and outcomes) statement as follows: population consisted of post-menopausal women (either natural or surgical); index was POI; control group included women with an age at menopause of 50–54 years; and the outcome of interest was composite and specific end-point of cardiovascular events.

Furthermore, the systematic review and meta-analysis was registered in the PROSPERO International Prospective Register of Systematic Reviews with the registration number CRD42022376480.

### 2.1 Eligibility Criteria

All types of analytic observational cohort studies assessing the risk of subsequent cardiovascular development in women with the diagnosis of POI were eligible to be included in this systematic review and meta-analysis. In addition, studies should have clearly defined POI (all, natural or surgical) and cardiovascular (CV) events; report the number or prevalence or hazard ratio or relative risk of CV events for quantifying the association of POI (menopause at <40 years) versus reference (menopause at 50–54 years) with cardiovascular events. The presence of preexisting CV events before POI diagnosis, presenting only CV risk factors such as dyslipidemia and diabetes mellitus, as well as those evaluating the composite CV events including CV risk factors and CV events, studies with patients having specific disorders related to POI such as genetic syndromes or Turner’s syndrome led to exclusion. Also, gray literature and non-original studies including reviews, commentaries, editorials, letters, meeting abstracts, case reports, conference proceedings, governmental or organizational reports, dissertations, theses, unpublished data and presentations that did not provide accurate and clear data on research variables were excluded.

### 2.2 Search Strategy

To find eligible studies published in scientific journals, a systematic literature search was carried out across three electronic databases of PubMed, Scopus, and Web of Science. The search spanned from Jan 1, 1990, to May 10, 2022 and involved combining relevant search terms (found in **Supplementary Table 1**) to narrow the search. (**Supplementary Table 1**). The search was limited to publications in English language and involving human subjects. Additionally, manual searches were performed on reference lists of selected studies and relevant reviews. The search strategies used in all databases were almost similar, with searches conducted based on titles, abstracts, and keywords.

### 2.3 Study Selection and Extraction

Two reviewers (SB-G and RB-Y) screened potentially relevant papers independently. Studies that did not meet the eligibility criteria based on their titles or abstracts were excluded, and the full text of the remaining studies was evaluated. Any discrepancies were resolved through discussion between the review authors or by appealing to the other team members if necessary. The extracted data included the study’s origin, publication year, country where the study was conducted, study duration, study population size, population characteristics (including age and body mass index (BMI)), outcome measurements (such as the number, prevalence, or risk of cardiovascular events), and were obtained from the included studies. To ensure the accuracy of data extraction and entry, the data was double-checked before the meta-analysis to avoid any potential bias.

### 2.4 Term Definition and Outcomes

POI was defined as a cessation of ovarian function before the age of 40 years. The primary outcome was the point prevalence and risk of the composite outcome of CV events and the secondary outcomes were the point prevalence and risk of specific CV events including coronary heart disease, death due to any cardiovascular event, pulmonary hypertension or hypertension in women with POI compared to women with menopausal age of 50–54 years.

### 2.5 Quality Appraisal

To assess the methodological quality and result presentation of the studies, the Newcastle-Ottawa scale (NOS) was employed [20]. This scale evaluates studies based on three criteria: participant selection (maximum of four stars), comparability of study groups (maximum of two stars), and assessment of outcome or exposure (maximum of three stars) for the outcome/exposure category. The results of the NOS evaluation can be found in **Supplementary Table 2**.

### 2.6 Statistical Analysis

The STATA software package (version 14; STATA Inc., College Station, TX, USA) was used to conduct statistical analysis. Heterogeneity was evaluated using the Chi-square test and *p*-value > 0.05 was interpreted as homogeneity. Publication bias was assessed using Begg’s or Egger tests as a formalized statistical test for statistically estimating funnel plot asymmetry to find any possible publication bias. In case of significant publication bias trim and fill method was used. The Pooled prevalence of outcomes of interest was applied using meta-prop method with pooled estimate after Freeman-Tukey Double Arcsine Transformation to stabilize the variances. Forest plots for each menopause group and by the subgroup of the CVD events were also illustrated. Pooled Incidence of CVD for POI group was also estimated per 1000 women. It was further adjusted for hormone therapy as well. Then, pooled hazard ratios were estimated by the subgroups of CVD events and also type of menopause. Sensitivity analysis was run to find influential studies, in the case. Sensitivity forest graphs visually provide the results, naming the omitted study on the left margin and presenting the resulting “omitted” meta-analytic summary statistics as a horizontal confidence interval and also the full, “combined” results are shown as solid vertical lines. An individual study is suspected of excessive influence if the point estimate of its “omitted” analysis lies outside the confidence interval of the “combined” analysis. *p*-value < 0.05 was set as statistically significant.

## 3. Results 

### 3.1 Systematic Search Results

**Fig. 1.**
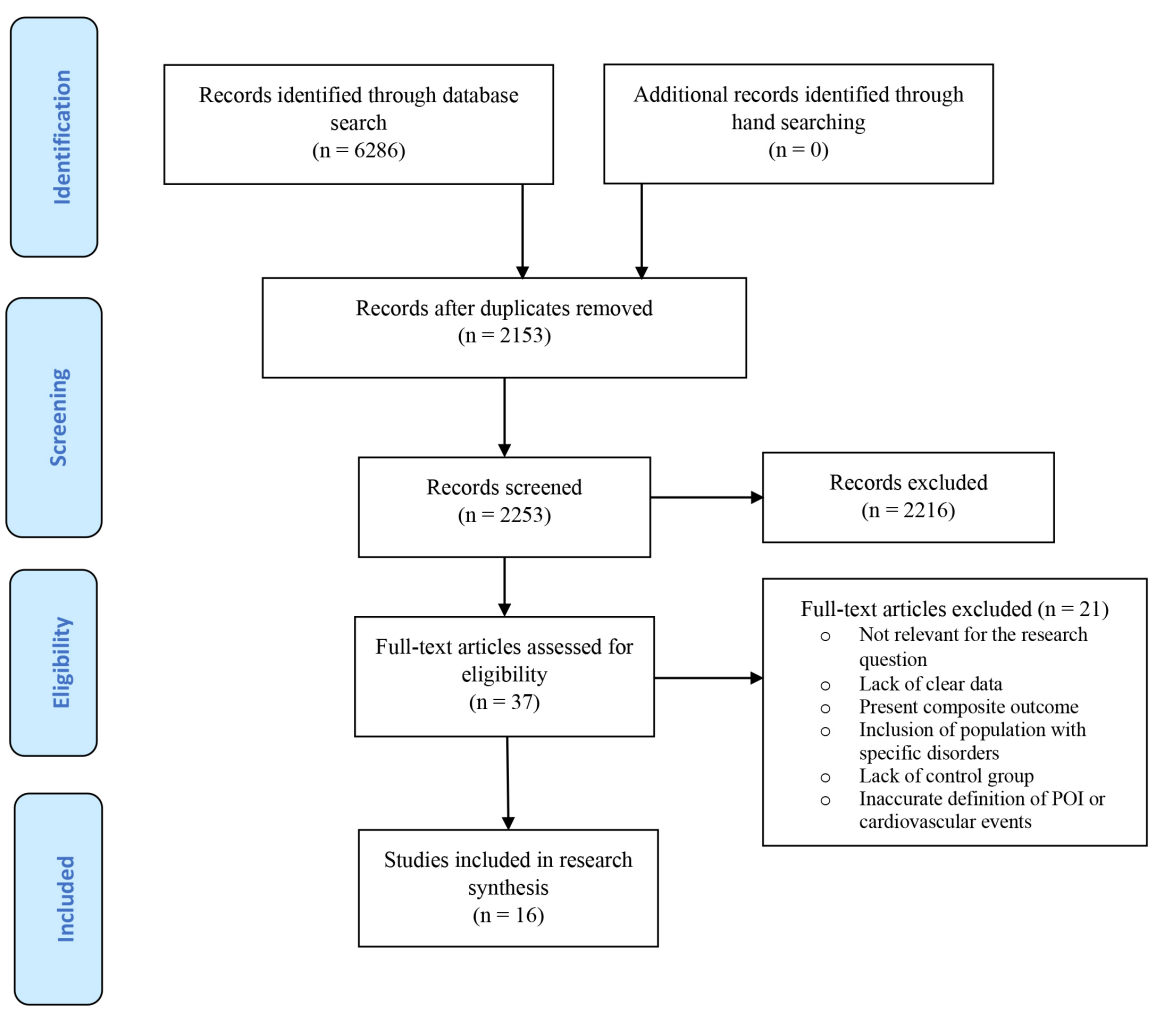
**The PRISMA flowchart for the search process**. POI, premature ovarian insufficiency.

The search yielded 6286 citations, including 2153 duplicates (Fig. 1). The screening of titles and abstracts resulted in the exclusion of 2216 studies. After a full-text appraisal of 37 studies, 16 studies were included [21, 22, 23, 24, 25, 26, 27, 28, 29, 30, 31, 32, 33, 34, 35, 36], involving 40,549 women who suffered from POI and 1,016,633 women as controls. Characteristics of these studies have been summarized in Table 1 (Ref. [21, 22, 23, 24, 25, 26, 27, 28, 29, 30, 31, 32, 33, 34, 35, 36]). A total of eight studies were conducted in European countries (including the Netherlands [34, 35, 36], UK [27, 28, 29], Denmark [33] and one in 10 European countries [24]), four studies in the USA [23, 30, 31, 32] and two studies in Asian countries (including Japan [21] and South-Korea [22, 26]).

**Table 1.** **Characteristics of the eligible studies included in the systematic review**.

First author, year	Country	Setting	POI group	Control group	Follow up time	Number of events	Number of events
			Sample size	Sample size		(Incidence %) in POI group	(Incidence %) in Control group
Baba* et al*. 2010 [21]	Japan	Jichi Medical School population-based prospective study	N = 237	N = 2171	Mean of 10.8 y	Stroke: 10 (4.2)	Stroke: 76 (3.5)
Choi* et al*. 2005 [22]	Korea	The Korean Elderly Pharmacoepidemiologic population-based Cohort study	N = 84	N = 2555	27,936 person-y (5 y)	Stroke: 3 (3.5)	Stroke: 87 (3.4)
Cooper* et al*. 1998 [23]	USA	National Health and Examination Survey Epidemiologic Follow-up study	N = 115	N = 1475	Mean of 4 y	Death due to any cardiovascular events: 9 (7.83)	Death due to any cardiovascular events: 71 (4.8)
Dam* et al*. 2019 [24]	10 European countries	EPIC-CVD, a case–cohort study, which includes data from 23 centres from 10 European countries	N = NM	N = NM	Median of 11 y	Coronary heart disease: 364 (43.8%)	Coronary heart disease: 1563 (45.4%)
Gallagher* et al*. 2011 [25]	China	Shanghai population-based Cohort study	N = 16,029	N = 450,359	Median of 10 y	Coronary heart disease: 7 (0.04)	Coronary heart disease: 175 (0.04)
						Stroke: 43 (0.27)	Stroke: 986 (0.22)
Hong* et al*. 2007 [26]	South Korean	Kangwha population-based Cohort study	N = 198	N = 948	Median of 15.8 y	Death due to any cardiovascular events: 27	Death due to any cardiovascular events: 101
Honigberg* et al*. 2021 [27]	UK	UK Biobank population-based Cohort study	N = 5201	N = 86,557	Median of 11.1 y	PH: 38 (0.73%)	PH: NM
							HR: 2.36, 95% CI: 1.49–3.73
Honigberg* et al*. 2019 [28]	UK	UK Biobank population-based Cohort study	N = 5548	N = 138,712	Median of 7 y	Coronary heart disease: 124 (2.24)	Coronary heart disease: 1646 (1.19)
						Heart failure: 50 (0.9)	Heart failure: 722 (0.5)
						Stroke: 35 (0.6)	Stroke: 584 (0.4)
						HTN: 301 (5.4)	HTN: 5007 (3.6)
						Heart valve disease: 44 (0.7)	Heart valve disease: 604 (0.4)
Honigberg* et al*. 2021 [29]	UK	UK Biobank population-based Cohort study	N = 1305	N = 18,301	Median of 7-13.1 y	Coronary heart disease: 162 (12.4)	Coronary heart disease 1568 (8.5)
Hu* et al*. 1999 [30]	USA	Nurses’ Health study, population-based Cohort study	N = NM	N = NM	Median of 18 y	Coronary heart disease: 18	Coronary heart disease: 375
						Stroke: 4	Stroke: 182
Ley* et al*. 2017 [31]	USA	Nurses’ Health study, population-based Cohort study	N = NM	N = NM	1,467,987 person-y	Coronary heart disease: 177	Coronary heart disease: 1306
						Stroke: 163	Stroke: 1239
Li* et al*. 2013 [32]	USA	Black Women’s Health study, population-based Cohort study	N = 586	N = 4747	Median of 13 y	Death due to any cardiovascular events: 15	Death due to any cardiovascular events: 70
Løkkegaard* et al*. 2006 [33]	Denmark	Danish Nurse	N = 380	N = 8186	Median of 5 y	Coronary heart disease: 7	Coronary heart disease: 3
		population-based Cohort study					HR: 2.1, 95% CI: 1.3–3.5
Ossewaarde* et al*. 2005 [34]	Netherlands	breast cancer screening cohort, population-based Cohort study	N = 454	N = 5753	Median of 17 y	Death due to any cardiovascular events: 50	Death due to any cardiovascular events: 445
van der Schouw* et al*. 1996 [35]	Netherlands	DOM project, population-based Cohort study	N = 459	N = 5741	Median of 20 y	Death due to any cardiovascular events: 43	Death due to any cardiovascular events: 391
Welten* et al*. 2021 [36]	Netherlands	(European Prospective Investigation into Cancer	N = 2407	N = 5353	Median of 15 y	Stroke: 121	Stroke: 310
		and Nutrition–Netherlands) population-based Cohort study					

N, number; NM, not mentioned; y, year. HR, hazard ratio; CI, confidence interval; POI, premature ovarian insufficiency; EPIC-CVD, European Prospective Investigation into Cancer and Nutrition (EPIC) study; PH, pulmonary hypertension; HTN, hypertension; DOM, Diacnostisch Onderzoek (investigation Mammacarcinoom).

This sample consisted of prospective studies with 4–20 years of follow up, which involved more than 33,000 women who suffered from POI and 730,850 women as controls. All studies reported the point prevalence of CV outcomes except two [30, 31], that presented the incidence of them. The studies reported on stroke (n = 4) [21, 22, 28, 36], death due to any cardiovascular events (n = 5) [14, 23, 32, 34, 35], coronary heart disease (n = 7) [24, 25, 28, 29, 30, 31, 33], pulmonary hypertension (n = 1) [27], heart failure (n = 1) [28], hypertension (n = 1) [28] and heart valve disease (n = 1) [28].

The quality appraisal of the included studies is reported in **Supplementary Table 2**. All studies were judged to have high and moderate quality and no studies had low quality.

**Fig. 2.**
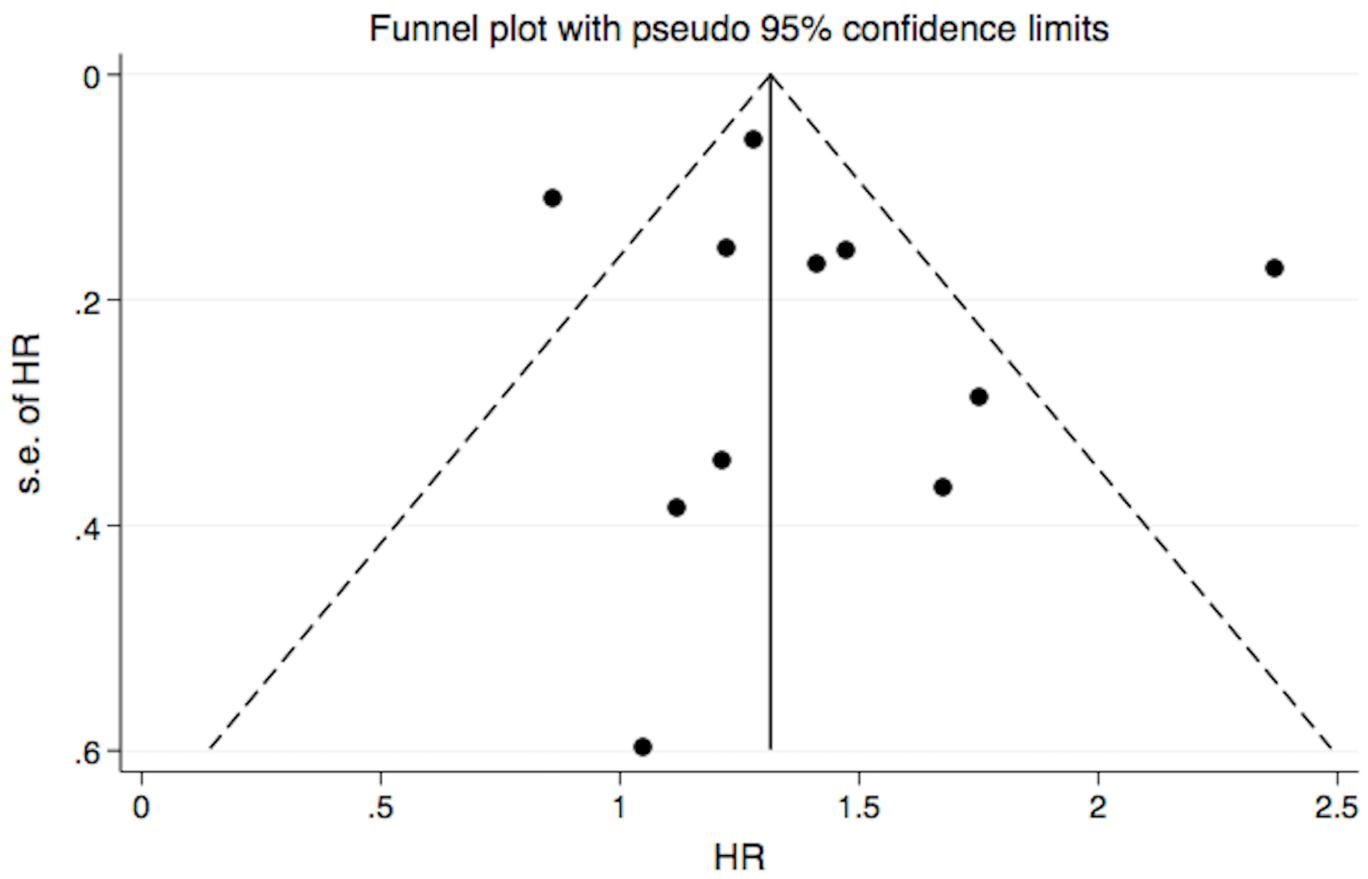
**Funnel plots exploring potential publication bias**. HR, hazard ratio.

Visual inspection of the funnel plot for CV events was symmetrical (Fig. 2), suggesting a low risk of publication bias, which was supported by the Egger test (*p* > 0.05). Due to a lack of data, we could not run funnel plot for individual studies.

### 3.2 Meta-Analyses of Primary Outcomes

**Fig. 3.**
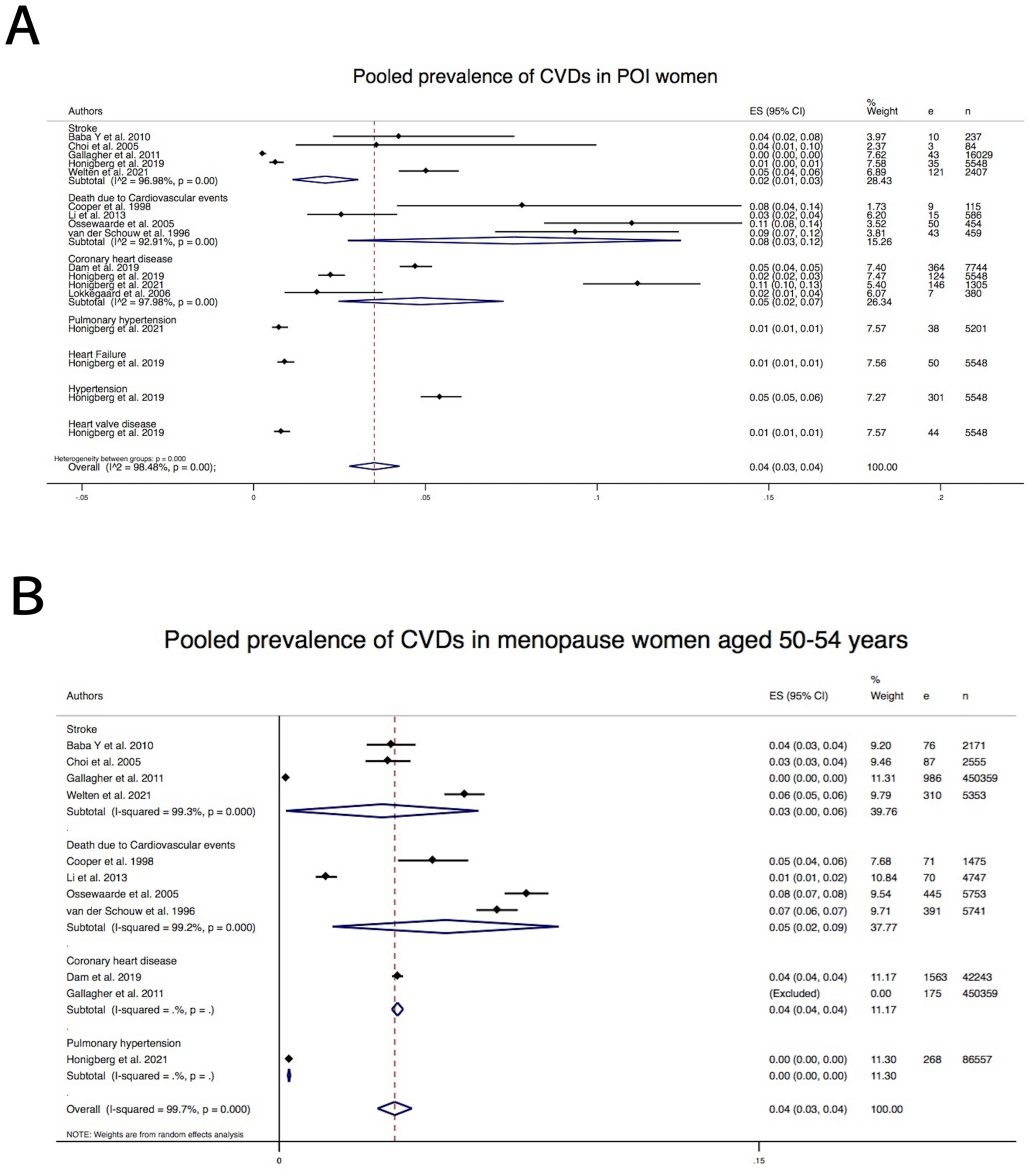
**Forest plot of pooled prevalence of cardiovascular events in premature ovarian failure (A) and Controls women with menopausal age of 50–54 years (B)**. (A) Pooled prevalence of cardiovascular events in premature ovarian insufficiency. (B) Pooled prevalence of cardiovascular events in controls women with menopausal age of 50–54 years. ES, effect size; e, event; n, number; CVDs, cardiovascular diseases; POI, premature ovarian insufficiency.

In terms of the composite outcome of CV events, a total of 16 studies involving 40,549 women with POI and 1,016,633 women with menopause aged 50–54 years were entered into the meta-analysis. The pooled prevalence of composite CV events in both groups of POI and controls, regardless of types of the CV events, were 4% (Pooled *p* = 4%, 95% CI: 3–4%, $I^{2}$: 98%, 40,549 women) and 4% (Pooled *p* = 4%, 95% CI: 3–4%, $I^{2}$: 99%, 1,016,633 women), respectively (and in the Forest plot, Fig. 3 and **Supplementary Table 3**).

**Fig. 4.**
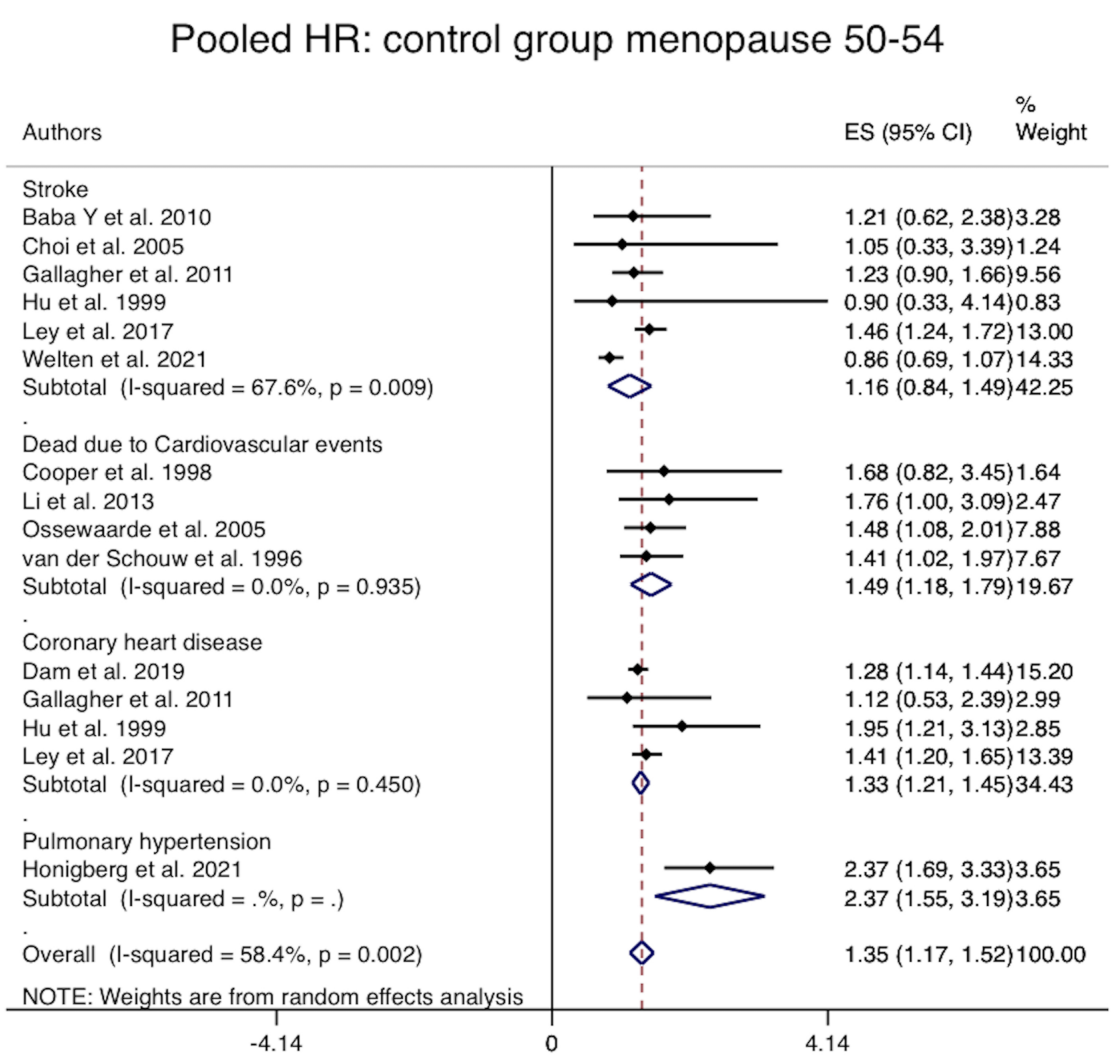
**Forest plot of the pooled risk of composite and individual cardiovascular events**. ES, effect size; HR, hazard ratio.

The pooled risk of composite CV events, regardless of the type of event, among women with the POI was significantly 1.4 fold higher than women with menopausal age 50–54 years (Pooled HR = 1.35, 95% CI: 1.17–1.52, $I^{2}$: 58%) (**Supplementary Table 3** and Fig. 4). However, results remain unchanged after adjustment for Hormone therapy (Pooled adjusted HR = 1.35, 95% CI: 1.06–1.63, $I^{2}$: 0%) (**Supplementary Fig. 1**)

### 3.3 Meta-Analyses of Secondary Outcomes

The pooled prevalence of specific CV events in women with POI and women with menopausal age of 50–54 years were 3% in both groups for stroke, 8% and 5% for death due to CV events respectively and 4% in both groups for coronary heart disease (**Supplementary Table 3** and Fig. 3).

In addition, subgroup analysis showed that the risk of death due to CV events and coronary heart disease in women with POI were 1.5 (Pooled unadjusted HR = 1.49, 95% CI: 1.18–1.79, $I^{2}$: 0%, 1614 women with POI and 17,716 with menopausal women for death) and 1.3 (Pooled unadjusted HR = 1.33, 95% CI: 1.21–1.45, $I^{2}$: 0%, 31,006 women with POI and 451,922 with 50–54 years menopausal age for coronary heart disease) fold higher than women with 50–54 years menopausal age, respectively. There was no difference between the groups regarding the risk of stroke (Pooled HR = 1.16, 95% CI: 0.84–1.49, $I^{2}$: 67%, 8276 women with POI and 460,438 with 50–54 years menopausal age), (Fig. 4 and **Supplementary Table 3**).

After adjustment for hormone therapy, the results for coronary heart disease remained unchanged (Pooled adjusted HR = 1.42, 95% CI: 1.17–1.66, $I^{2}$: 0%), but, the previously observed significant level of risk of death due to CV events was no longer statistically significant (Pooled adjusted HR = 1.22, 95% CI: 0.74–1.70, $I^{2}$: 75.3%), (**Supplementary Fig. 1**).

### 3.4 Sub-Group Analysis

**Fig. 5.**
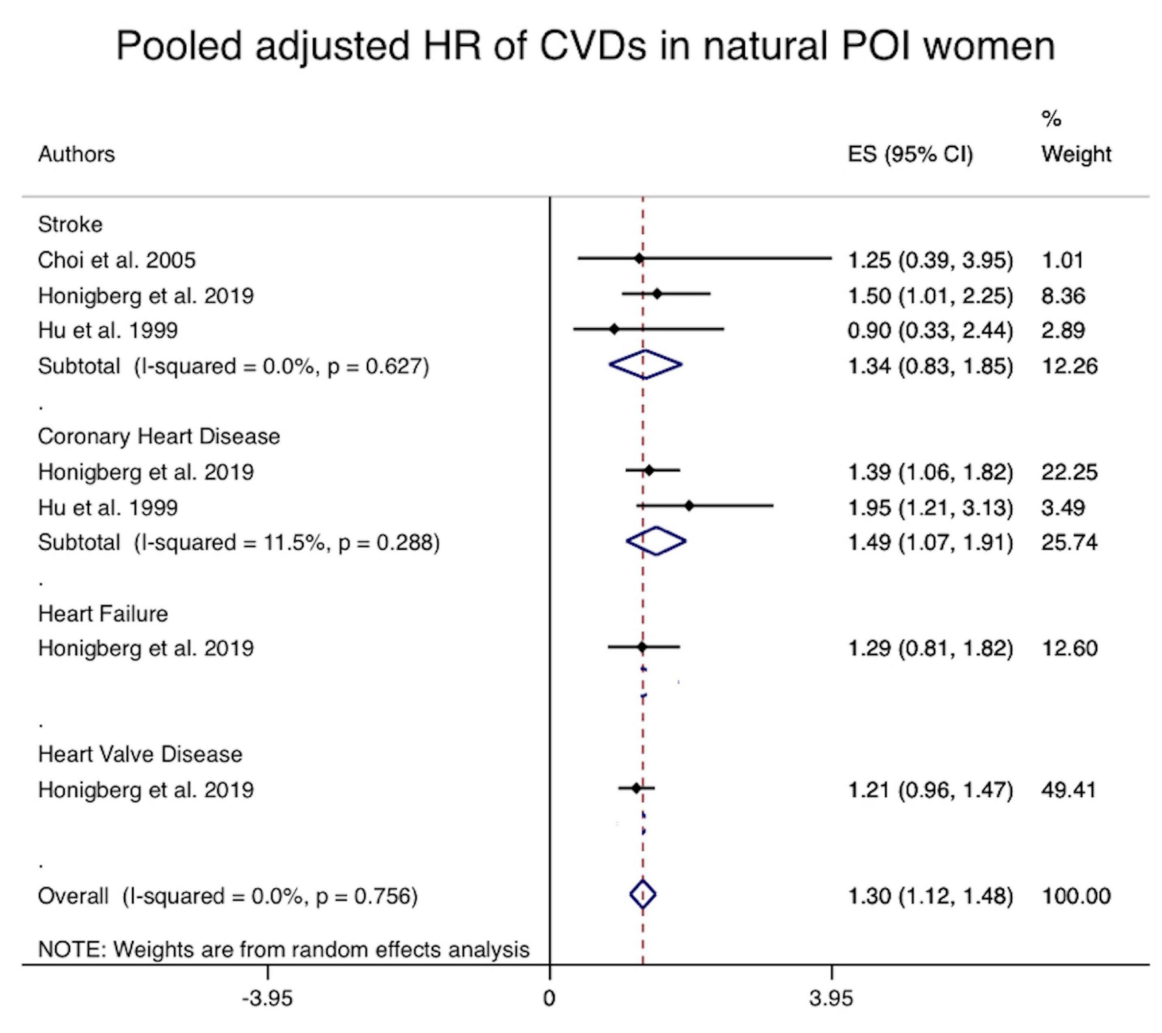
**Forest plot of the pooled adjusted risk of composite and individual cardiovascular events**. ES, effect size; HR, hazard ratio; CVDs, cardiovascular diseases; POI, premature ovarian insufficiency.

The pooled adjusted risk of composite CV events and also coronary heart disease, among sub group of women with natural POI were significantly 1.3 (Pooled adjusted HR = 1.30, 95% CI: 1.12–1.48, $I^{2}$: 0%) and 1.4 (Pooled adjusted HR = 1.49, 95% CI: 1.07–1.91, $I^{2}$: 0%) fold higher than women with natural menopause age 50–54 years. There was no difference between the groups regarding the risk of stroke in subgroup of women with natural POI compared to women with menopause age 50–54 years. There was not sufficient data for pooled analysis of death due to CV events and other specific CV events in this subgroup (Fig. 5).

Data on potential confounding factors were limited. In addition, there was not sufficient data for pooled analysis of other specific CV events including heart failure, heart valve disease, hypertension and pulmonary hypertension.

Results of sensitivity analyses showed that no single study essentially changed the pooled prevalence and risk of all outcomes (**Supplementary Fig. 2A–C**). However, the number of studies for specific outcomes were too small to assess funnel plot asymmetry reliably.

## 4. Discussion

The present systematic review and meta-analysis of observational prospective studies with 4–20 years of follow up revealed that POI is an important risk factor for composite CV events, particularly for coronary heart disease after adjustment for hormone therapy. However, no difference was found regarding the risk of stroke and death due to CV events in POI and women experiencing menopause at 50–54 years. Subgroup analysis among those experienced natural POI confirmed these findings. We found no heterogeneity and publication bias among the included studies.

Cardiovascular disease is one of the leading causes of mortality among women [37]. There is substantial evidence indicating that the risk significantly rises following menopause [38, 39]. While the specific mechanisms behind the development of cardiovascular disease in menopausal women are not yet fully understood, several possibilities have been suggested. One potential mechanism is that the reduced exposure to endogenous estrogens — which have a protective effect on the cardiovascular system — may play a role. Menopause, characterized by estrogen deprivation, can lead to increased secretion of substances that contribute to oxidative stress and vasoconstriction, ultimately impairing endothelial function [40, 41]. Moreover, research has shown that menopausal transition is associated with negative changes in β-cell function and insulin sensitivity, serum lipid levels, basal metabolic rate, and body composition (including the deposition of fat around the heart) [42, 43]. These factors may represent early steps in the development of cardiovascular events in postmenopausal women.

POI is considered the most severe form of early menopause [16]. Although previous studies have demonstrated that early menopause is an independent risk factor for cardiovascular events [13, 44, 45] there is limited conclusive evidence regarding POI and its association with cardiovascular events. In agreement with our findings, Roeters van Lennep *et al*. [18] in a systematic review and meta-analysis of 10 observational studies published until 2012, reported that POI was significantly associated with an increased risk of developing overall cardiovascular disease (HR: 1.61, 95% CI 1.22–2.12) and ischemic heart disease (HR: 1.69, 95% CI 1.29–2.21). However, the results of that meta-analysis should be interpreted with caution since two of the ten studies with high weighting in the final pooled result [12, 46], did not strictly follow the definition of POI and included menopausal women aged 40 years as POI.

Another plausible explanation of the positive association between POI and CV events could be related to the fact that POI is associated with other cardio-metabolic disorders. Recently, Anagnostis *et al*. (2019) [42] in a meta-analysis of thirteen studies reported that both early menopause >45 years and POI are significantly associated with increased risk of type 2 diabetes (OR: 1.15, 95% CI: 1.04–1.26, *p* = 0.003; and OR: 1.50, 95% CI: 1.03–2.19, *p* = 0.033) respectively. In another meta-analysis of 21 individual studies, Cai *et al*. (2022) [47] demonstrated that POI patients presented significantly higher waist circumference, total cholesterol, low-density lipoprotein, high-density lipoprotein, triglycerides, and fasting glucose. We hypothesize that the co-existence of other cardio-metabolic risk factors may predispose women with POI to CV events.

However, since underlying reasons and mechanisms related to surgical menopause as a complete lack of ovarian activity due to surgical removal of the organs, could be different from those who experienced natural POI, we performed a subgroup analysis based on the type of POI, whether natural or surgical. The results were found to be similar to the overall outcomes. It is worth mentioning that a considerable proportion of the study participants in this meta-analysis experienced natural POI, it was reasonable to anticipate that the outcomes would not deviate significantly.

Additionally, could highlight the need for the lack of comprehensive long-term studies including RCTs on POI. An international registry for women with POI could be a valuable resource for understanding the natural history of the condition and the long-term health outcomes associated with POI. Such a registry could collect data on a large number of women with POI, including demographic and clinical characteristics, as well as information on treatment and health outcomes over time. The data collected from such a registry could potentially help to fill the gap in evidence regarding the long-term health outcomes of women with POI, as well as provide valuable information for future research studies. In addition, a registry could serve as a resource for clinicians and researchers to improve patient care, support guideline development, and identify research priorities.

The main clinical implication of the current meta-analysis is to identify women with POI as a high risk population for CV events. It needs to be clearly defined if earlier medical therapy such as exogenous estrogen or lifestyle interventions would be valuable compared with the general population [48, 49] as well.

## 5. Conclusions

In conclusion, POI is associated with an increased risk of CV events, particularly coronary heart disease. Our findings also extend prior work with data supporting POI as a risk-enhancing factor for CV events. Future studies are warranted to confirm these findings and to explore the potential underlying mechanism linking CV events and POI.

## 6. Strengths and Limitations

Our study had certain limitations, including a relatively small number of studies included in the meta-analysis. Additionally, due to the lack of information in individual included studies, our results did not adjust for potential confounders such as women’s lifestyle, obstetrics history, age, and lipid profile. Additionally, it is suggested that the type of menopause including natural and surgical menopause may affect cardiometabolic disturbances [50]. However, this factor was not evaluated in most included studies. Besides, the menopausal age was self-reported in included studies. But it was argued that self-report could be a valid data collection tool for age at menopause and suggested that women could provide data on their natural or surgical menopausal age with sufficient accuracy [51, 52]. Further, some included studies published more than 20 years ago, which need to be updated in future studies. Although narrow inclusion criteria for this study led to the adoption of a small number of studies to this meta-analysis, the findings of our meta-analysis comprehensively add new knowledge to the body of international literature and also help with the provision of updated evidence on this important topic. Besides, in our present meta-analysis, we adhered to the precise definition of POI which led to the adoption of high quality evidence and a present precise presentation of the results. Moreover, it is worth noting that all of the included studies in the current meta-analysis had a population-based design, as a representative of general population characteristics with minimizing the selection bias, therefore the finding of this study could be extrapolated to the general population.

## Data Availability

Data are available on requested.

## Abbreviations

CV, cardiovascular; POI, premature ovarian insufficiency; HR, hazard ratio; CI, confidence interval.

## Supplementary Material

Supplementary material associated with this article can be found, in the online version, at https://doi.org/10.31083/j.rcm2407193.
