# Supplementary material for: Cardiovascular Events Among Women with Premature Ovarian Insufficiency: A Systematic Review and Meta-Analysis
Source: Rev Cardiovasc Med. 2023 Jul 4;24(7):193. doi: 10.31083/j.rcm2407193 (PMC11266470; doi:10.31083/j.rcm2407193)
Supplement: Supplementary file 1 [file 2153-8174-24-7-193-s1.zip › Supplementary Material.docx]

Supplementary table 1. Key words used for search in this study

| (cardiovascular OR “cardiovascular disease” OR “cardiovascular events” OR “myocardial infarction” OR MI OR “Coronary heart disease” OR IHD OR “Ischemic heart disease” OR Angina OR “heart attack” OR CAD OR “coronary arterial disease” OR Stroke OR “Cerebral Infarction” OR “hemorrhagic stroke” OR “ischemic stroke” OR CVD OR “Cerebrovascular disease” OR “Heart failure” OR “Heart valve disease” OR “Aortic stenosis” OR “Mitral regurgitation” OR “cardiovascular dead” OR “cardiovascular mortality” OR “cardiovascular morbidity” OR PH OR “Pulmonary hypertension” OR HTN Or hypertension) AND (“premature ovarian insufficiency” OR POI OR “Premature Ovarian failure” OR POF OR “primary ovarian insufficiency” OR “ovarian insufficiency” OR menopause Or “early menopause” OR climacteric) |
| --- |

Supplementary table 2: Quality assessment of the included studies using the Newcastle–Ottawa Quality Assessment Scale for cohort studies.

|  | **SELECTION** | | | | **COMPARABILITY** | **Outcome** | | | **Total scores** |
| --- | --- | --- | --- | --- | --- | --- | --- | --- | --- |
| **Author, Year** | Representativeness of the exposed cohort * | Selection of the non-exposed cohort * | Ascertainment of exposure * | No outcome of interest at the start of study * | A: Study controls for age and/or BMI *  B: Study controls for *other confounders | A: doctor’s diagnosis OR objective measurements  B: parent/self-reported * doctor’s diagnosis OR use of medication | Follow-up long enough for outcomes * | Adequacy of follow up of cohorts * |  |
| Baba et al. 2010 ^19^ | * | * | * | * | ** | * | * | * | 9* |
| Choi et al. 2005 ^38^ | * | * | * | * | ** | * | - | * | 8* |
| Cooper et al. 1998 ^44^ | * | * | * | * | ** | * | - | * | 8* |
| Dam et al. 2019 ^49^ | * | * | * | * | ** | * | * | * | 9* |
| Gallagher et al. 2011 ^65^ | * | * | * | * | * | * | * | * | 8* |
| Hong et al. 2007 ^79^ | * | * | * | * | ** | * | * | * | 9* |
| Honigberg et al. 2021 ^81^ | * | * | * | * | ** | * | * | * | 9* |
| Honigberg et al. 2019 ^82^ | * | * | * | * | ** | * | - | * | 8* |
| Honigberg et al. 2021 ^83^ | * | * | * | * | ** | * | * | * | 9* |
| Hu et al. 1999 ^85^ | * | * | * | * | ** | * | * | * | 9* |
| Ley et al. 2017 ^118^ | * | * | * | * | ** | * | * | * | 9* |
| Li et al. 2013 ^119^ | * | * | * | * | ** | * | * | * | 9* |
| Løkkegaard et al. 2006 ^132^ | * | * | * | * | ** | * | - | * | 8* |
| Ossewaarde et al. 2005 ^151^ | * | * | * | * | * | * | * | * | 8* |
| van der Schouw et al. 1996 ^204^ | * |  |  | * | ** | * | * | * | 7* |
| Welten et al. 2021 ^214^ | * | * | * | * | ** | * | * | * | 9* |

Supplementary table 3. Meta-analysis of included studies conducted on the prevalence of cardiovascular events and association with premature ovarian insufficiency

| Outcomes | | POI population (menopausal age less than 40 years) | | | | Controls (menopausal age 50-54 years) | | | | Hazard Ratio ^€^ | |
| --- | --- | --- | --- | --- | --- | --- | --- | --- | --- | --- | --- |
|  |  | Number | | Prevalence | | Number | | Prevalence | |  | |
|  |  | Study | Women | I^2^, % | Pooled P% (95%, CI%) | Studies | Women | I^2^, % | Pooled P% (95% CI%) | I^2^, % | Pooled HR (95% CI) |
| Composite CV events | | 14* | 40,549 | 98 | 4 (3-4) | 10 | 1016633 | 99.7 | 4 (3-4) | 58 | **1.35 (1.17-1.52)** |
| Individual CV events | | | | | | | | | | | |
|  | Stroke | 4 | 8,276 | 97 | 3.30 (0.22-6.37) | 4 | 460438 | 99.3 | 3.22 (021-6.23) | 67 | 1.16 (084-1.49) |
|  | Death due to CV events | 4 | 1,614 | 92.9 | 7.59 (2.47-12.44) | 4 | 17716 | 99.2 | 5.20 (1.67-8.73) | 0 | **1.49 (1.18-1.79)** |
|  | Coronary heart disease | 5 | 31,006 | 99.4 | 3.89 (1.49-6.29) | 2 | 451922 | 0 | 3.7 (3.52-3.88) | 0 | **1.33 (1.21-1.45)** |
|  | Pulmonary hypertension | 1 | 5221 | -** | -** | 1 | 86557 | -** | -** | -** | -** |
|  | hypertension | 1 | 5548 | -** | -** | - | -** | -** | -** | -** | -** |
|  | Heart failure | 1 | 5548 | -** | -** | - | -** | -** | -** | -** | -** |
|  | Heart valve disease | 1 | 5548 | -** | -** | - | -** | -** | -** | -** | -** |
| CV: cardiovascular; I^2^: I-squared;  ^€^ Bold values indicates significant result  *Two studies of (Hu et al. 1999) and (Ley et al. 2017) did not included in the meta-analysis of pooled prevalence, since they reported incidence.  ** Lack of sufficient data | | | | | | | | | | | |

Supplementary figure 1. Forest plot of the pooled risk of composite and individual cardiovascular events adjusted for hormone therapy.


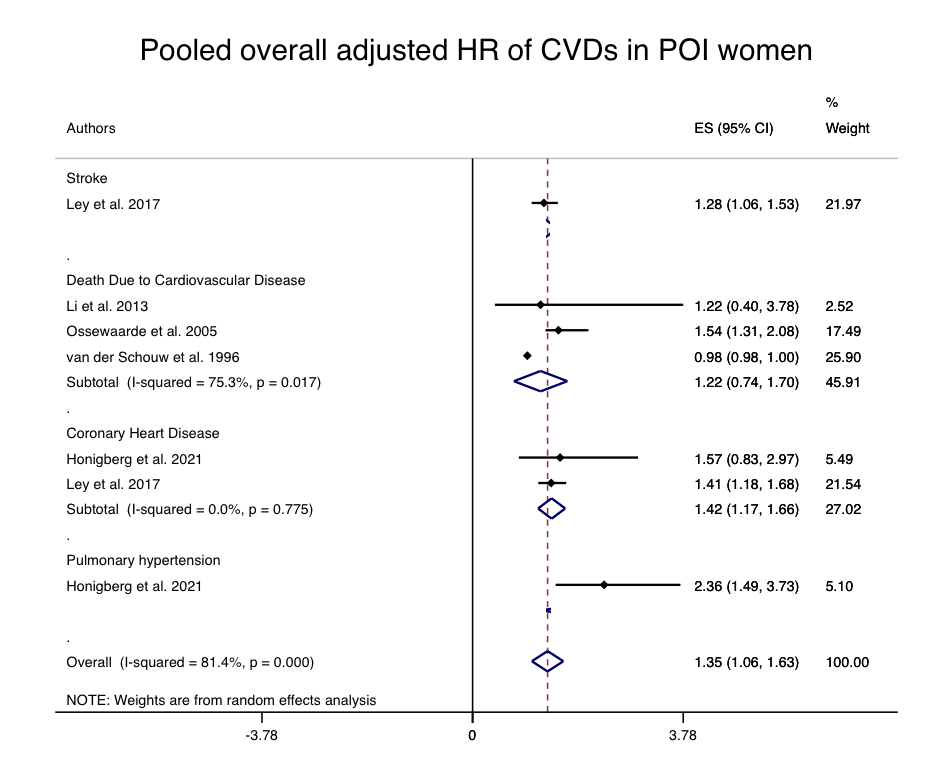


Supplementary figure 2. Sensitivity analysis

A: Sensitivity analysis of pooled prevalence of cardiovascular events in premature ovarian failure

B: Sensitivity analysis of pooled prevalence of cardiovascular events among controls women with menopausal age of 50-54 years

C: Sensitivity analysis of pooled hazard of cardiovascular events among POI vs. controls women with menopausal age of 50-54 years
